# Detection of Ultra-Rare Mitochondrial Mutations in Breast Stem Cells by Duplex Sequencing

**DOI:** 10.1371/journal.pone.0136216

**Published:** 2015-08-25

**Authors:** Eun Hyun Ahn, Kensen Hirohata, Brendan F. Kohrn, Edward J. Fox, Chia-Cheng Chang, Lawrence A. Loeb

**Affiliations:** 1 Department of Pathology, University of Washington, Seattle, Washington, United States of America; 2 Institute of Stem Cell and Regenerative Medicine, University of Washington, Seattle, Washington, United States of America; 3 Department of Biochemistry, University of Washington, Seattle, Washington, United States of America; 4 Department of Pediatrics and Human Development, Michigan State University, East Lansing, Michigan, United States of America; Institut Pasteur, FRANCE

## Abstract

Long-lived adult stem cells could accumulate non-repaired DNA damage or mutations that increase the risk of tumor formation. To date, studies on mutations in stem cells have concentrated on clonal (homoplasmic) mutations and have not focused on rarely occurring stochastic mutations that may accumulate during stem cell dormancy. A major challenge in investigating these rare mutations is that conventional next generation sequencing (NGS) methods have high error rates. We have established a new method termed Duplex Sequencing (DS), which detects mutations with unprecedented accuracy. We present a comprehensive analysis of mitochondrial DNA mutations in human breast normal stem cells and non-stem cells using DS. The vast majority of mutations occur at low frequency and are not detectable by NGS. The most prevalent point mutation types are the C>T/G>A and A>G/T>C transitions. The mutations exhibit a strand bias with higher prevalence of G>A, T>C, and A>C mutations on the light strand of the mitochondrial genome. The overall rare mutation frequency is significantly lower in stem cells than in the corresponding non-stem cells. We have identified common and unique non-homoplasmic mutations between non-stem and stem cells that include new mutations which have not been reported previously. Four mutations found within the MT-ND5 gene (m.12684G>A, m.12705C>T, m.13095T>C, m.13105A>G) are present in all groups of stem and non-stem cells. Two mutations (m.8567T>C, m.10547C>G) are found only in non-stem cells. This first genome-wide analysis of mitochondrial DNA mutations may aid in characterizing human breast normal epithelial cells and serve as a reference for cancer stem cell mutation profiles.

## Introduction

Stem cells, like other cells within tissues, incur endogenous and environmental DNA damage, which, if not repaired, can result in mutations. The ability of stem cells to differentiate and their capacity to renew tissues present promising new approaches in regenerative medicine, tissue engineering, and biotechnology. However, mutation accumulation in stem cells or expanded populations of partially differentiated stem cells can increase the risk of tumor formation [1–3]. Unrepaired DNA lesions, accumulated during a person’s lifespan, could result in incorporation of non-complementary nucleotides (mutations). It is also conceivable that the accumulation of tissue-specific mutations promotes transcription of specific sets of genes that are required for differentiation. To our knowledge, no study has reported the mutation status of the whole mitochondrial DNA in human normal stem cells.

The human genome is divided into a large nuclear genome encoding more than 20,000 genes and a small circular mitochondrial (mt) genome encoding 37 genes [4]. Each mitochondrion contains many copies of a small circular DNA molecules (16569 bases) and each cell has many hundred to thousand copies of mtDNA. Mitochondrial DNA is vulnerable to reactive oxygen species (ROS)-mediated damage and is believed to be more prone to accumulating mutations than are nuclear DNA. This is likely due to, in part, their close proximity to the electron transport chain, their lack of protective histones, and relatively limited DNA repair capacity [5,6].

The advent of Duplex Sequencing (DS) [7–9] has made it feasible for us to accurately analyze mutations of the entire complement of mtDNA present in cells. DS is found to be >10,000-fold more accurate than conventional next generation sequencing (NGS) [7,9,10]. Unlike conventional sequencing technologies that sequence only a single strand of DNA, DS sequences both strands of DNA and importantly, only scores mutations if the mutations are present as complementary substitutions in both strands of a single DNA molecule. By comparison, conventional NGS methods can reliably detect only clonal mutations (homoplasmic variants) due to high background error frequency (10^−2^ to 10^−3^) [10,11]. In contrast, DS, with its extremely low background error frequency (<5x10^-8^), enables the detection of rarely occurring mutations. In the present study, we have classified mutations (variants) into homoplasmic (95–100%), high-heteroplasmic (>20 to <95%), low-heteroplasmic (>0.5 to 20%), and rare (0.5% or less) variants based on their percentages of prevalence at the same mitochondrial genome locations. Maternally inherited mitochondrial mutations or variants arising during early embryonic development are more likely to be homoplasmic/clonal (i.e., the same mutation existing at the same genome location in all or most mtDNA molecules). We have divided stochastic variants into rare and low-heteroplasmic variants. We focused our analysis on rare variants and low-heteroplasmic variants as they most likely represent *de novo* somatic variants.

We present an ultra-deep mutation analysis for the whole mtDNA genome in human breast normal epithelial cells (non-stem *vs*. stem) by utilizing Duplex Sequencing. Our results indicate that stochastic rare mutation frequency is lower in stem cells than in the corresponding non-stem cells. This suggests that mitochondrial genome is maintained with greater fidelity in stem cells than in non-stem cells.

## Results

Normal cells were isolated from breast tissues of three independent healthy women (ID # 11, #30, and #31). Then, the breast epithelial cells with stem cell features (referred to as ‘stem cells’ hereinafter) and without stem cells features (‘non-stem cells’) were separated, cultured, and characterized as we had reported previously [12–15]. We determined homoplasmic variants as well as heteroplasmic and rare variants throughout the whole mitochondrial genome using Duplex Sequencing.

### Duplex Sequencing (DS) data yield and mutation frequencies of SSCS and DCS

The frequency of mutations (variants) in the mitochondrial genome was determined on DNA isolated from human breast normal epithelial cells. DNA libraries were prepared and analyzed for DS. With the DS method, we sequence the two complementary strands of each DNA molecule. In doing so, we generate two single strand consensus sequences (SSCS) and a duplex consensus sequence (DCS). The average number of nucleotides sequenced at each genome position (depth of SSCS) for all samples were ~7000 (S1 Table). The DCS, assembled by pairing complementary SSCS with each other, covered an average depth of ~1500 for all samples. The rare mutation frequency of SSCS is about 4.6-fold higher by average than that of DCS (S1 Table), demonstrating the ability of DS to eliminate artifactual errors that are scored by sequencing techniques that do not make use of the inherent double-strand feature of DNA.

### Stem cells show significantly lower frequencies of rare mutations than the corresponding rare mutations in non-stem cells

**Fig 1:**
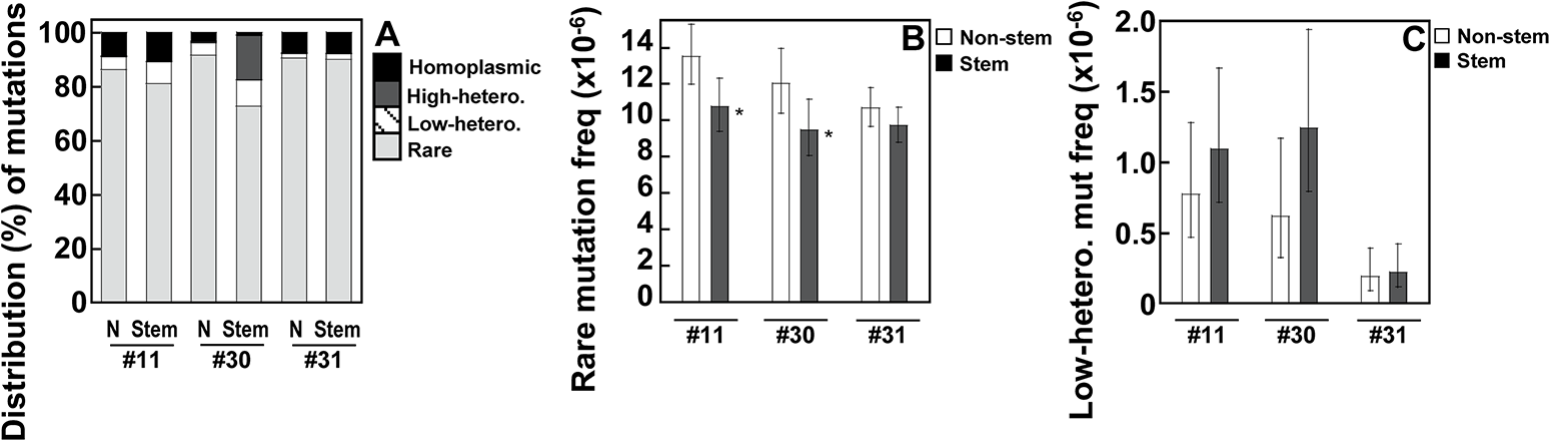
Non-homoplasmic mutation frequency in the whole mtDNA. Point mutations in the whole mtDNA were determined using DS. Data are from human breast normal epithelial cells (non-stem (N) *vs*. stem) developed from women (ID #11, #30, and #31). (A-B) The cutoffs of mutation frequency (% clonality) used for rare, low-heteroplasmic, high-heteroplasmic, and homoplasmic mutations are: 0−0.5%, >0.5−20%, >20−<95%, and 95−100%, respectively. (A) The distribution (%) of rare, low-heteroplasmic, high-heteroplasmic, and homoplasmic mutations is calculated as numbers of corresponding specific-clonality range mutations per numbers of total (0–100% clonality) mutations. (B-C) Error bars represent the Wilson Score 95% confidence intervals. (B) Significant differences in rare mutation frequencies between non-stem and stem cells from two women (ID #11 and #30) are indicated (*p* <0.05 (^*^) by the 2-sample test for equality of proportions with continuity correction).

Most point mutations are found at less than 0.5% clonality (Fig 1A) and thus would not be accurately scored by conventional NGS. However, these rare variants are accurately detected by Duplex Sequencing. In two (women ID #11 and #30) of the three paired groups of normal cells, the frequencies of rare mutations in stem cells is significantly lower than in those of non-stem cells (Fig 1B: *p*-values 0.015 and 0.037 for ID #11 and #30, respectively). In contrast, there is no significant difference between the frequencies of mutations greater than 0.5% (Fig 1C).

### The most prevalent point mutation types are C>T/G>A and A>G/T>C transitions and a strand-bias exists

**Fig 2:**
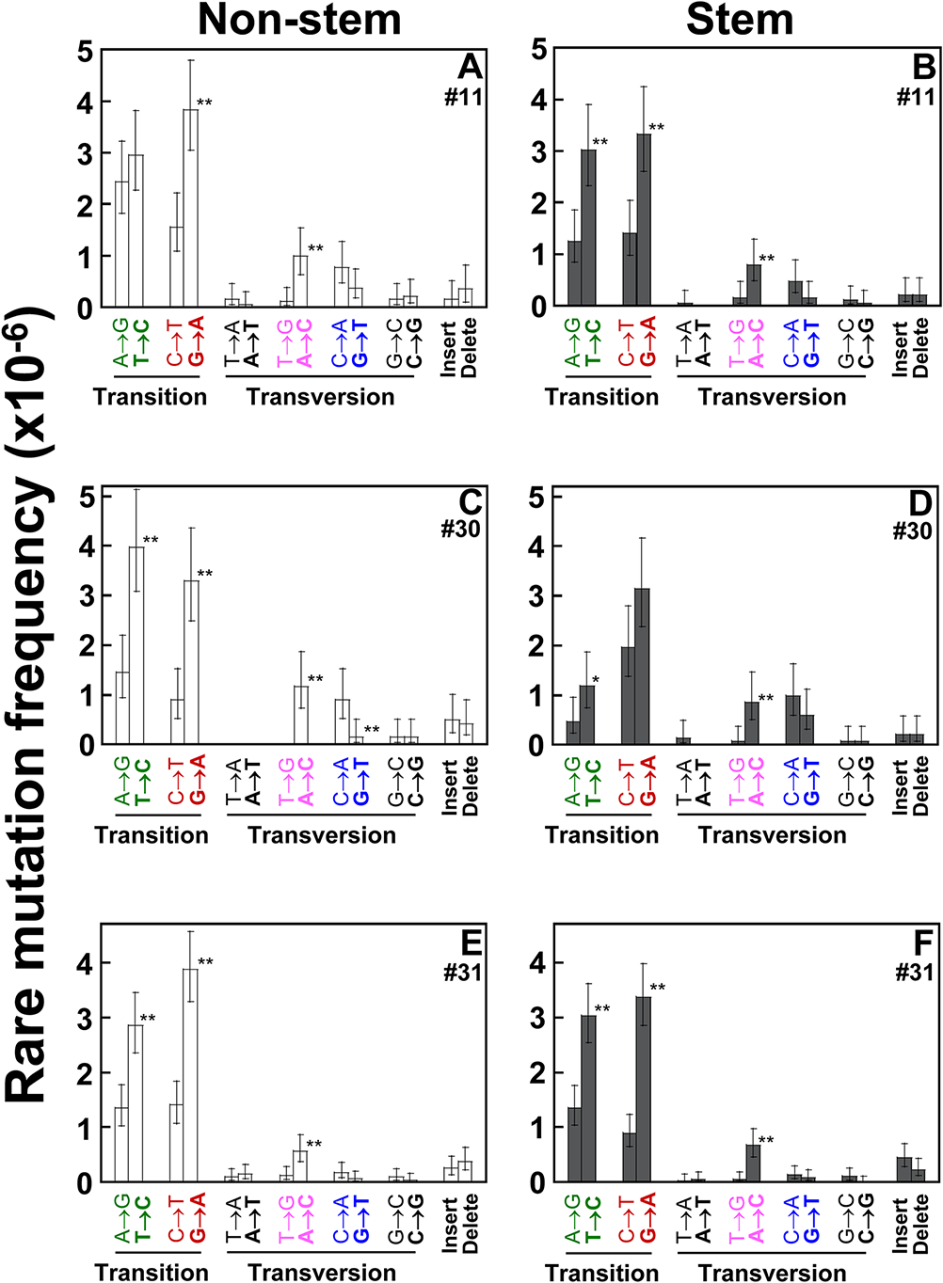
Frequencies of each type of rare mutations in the whole mtDNA. Types of rare point mutations and insertions and deletions (INDELs) in the whole mtDNA were determined using DS. Data are from human breast normal epithelial cells (non-stem *vs*. stem) developed from women (ID #11, #30, and #31). Error bars represent the Wilson Score 95% confidence intervals. Significant differences in mutation frequencies between the two groups are indicated (*p* <0.005 (^**^) by the 2-sample test for equality of proportions with continuity correction).

Duplex Sequencing enables the identification of 12 point mutation (substitution) types as well as insertions and deletions (INDELs). The A>G/T>C and C>T/G>A transitions are the most prevalent types of rare mutations (Fig 2A–2F). INDELs are found infrequently.

Human mitochondrial (mt) genome encodes 37 mt genes (22 tRNAs, 2 rRNAs, and 13 proteins-coding genes), with only less than 7% of the sequence considered non-coding [16,17]. Two strands of mtDNA are composed of heavy (H) and light (L) strands [18]. Our sequencing data are referenced to the L-strand. On the L-strand, G>A mutations are significantly more prevalent than C>T (Fig 2A–2C, 2E and 2F), T>C mutations are significantly more prevalent than A>G (Fig 2B–2F), and A>C mutations are significantly more prevalent than T>G (Fig 2A–2F). This higher prevalence of G>A, T>C, and A>C mutations on the L-strand indicates a significant strand orientation bias of human breast mtDNA.

**Fig 3:**
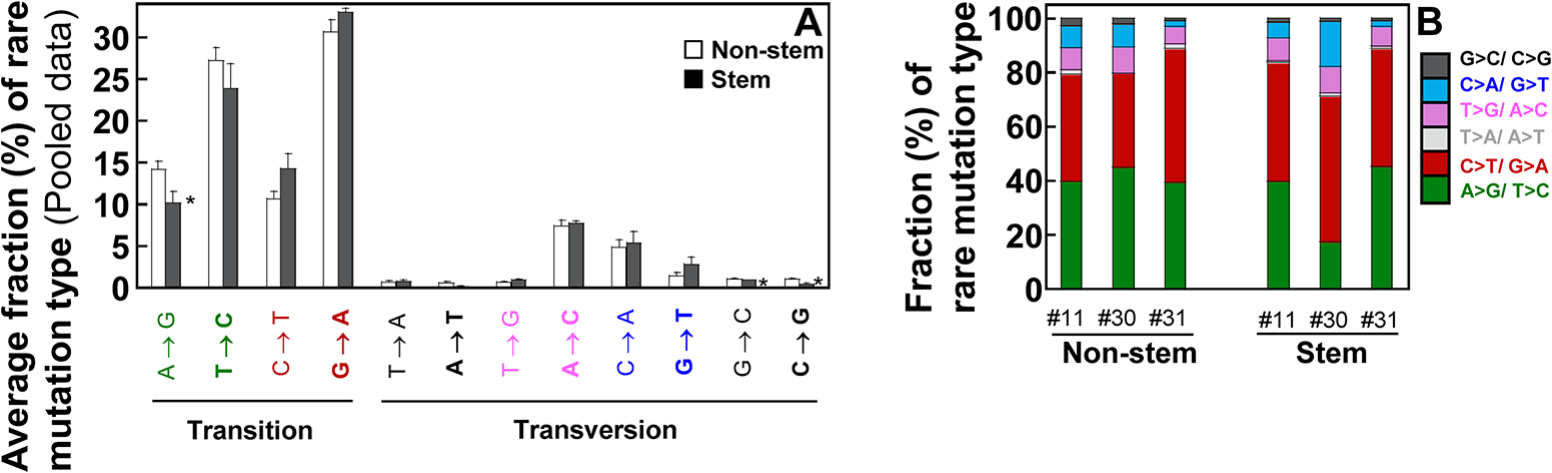
Fraction (%) of each type of rare mutations in the whole mtDNA. Types of rare point mutations in the whole mtDNA were determined using DS. (A) Data (mean ± SEM) are pooled from women (ID #11, #30, and #31). Significant differences in fractions (%) of mutation types between the two groups are indicated (*p* <0.05 (^*^) by Mann-Whitney U-test).

To compare the distribution of 12 mutation types between the two cell types, each mutation type of cells pooled from all three women is quantitated as a percentage (%) of overall rare mutations (Fig 3A). The fractions (%) of A>G, G>C, and C>G mutations are significantly lower in stem cells than in non-stem cells (*p* = 0.049 by Mann-Whitney U test), while percentages of other mutation types are not significantly different between the two cell types. The 12 mutation types are consolidated into 6 mutation types by grouping with complementary sequences and each mutation type is further presented as a percentage (%) of overall rare mutations for each set of independent normal cells (Fig 3B).

### Neighboring bases influence the frequencies and types of rare mutations

**Fig 4:**
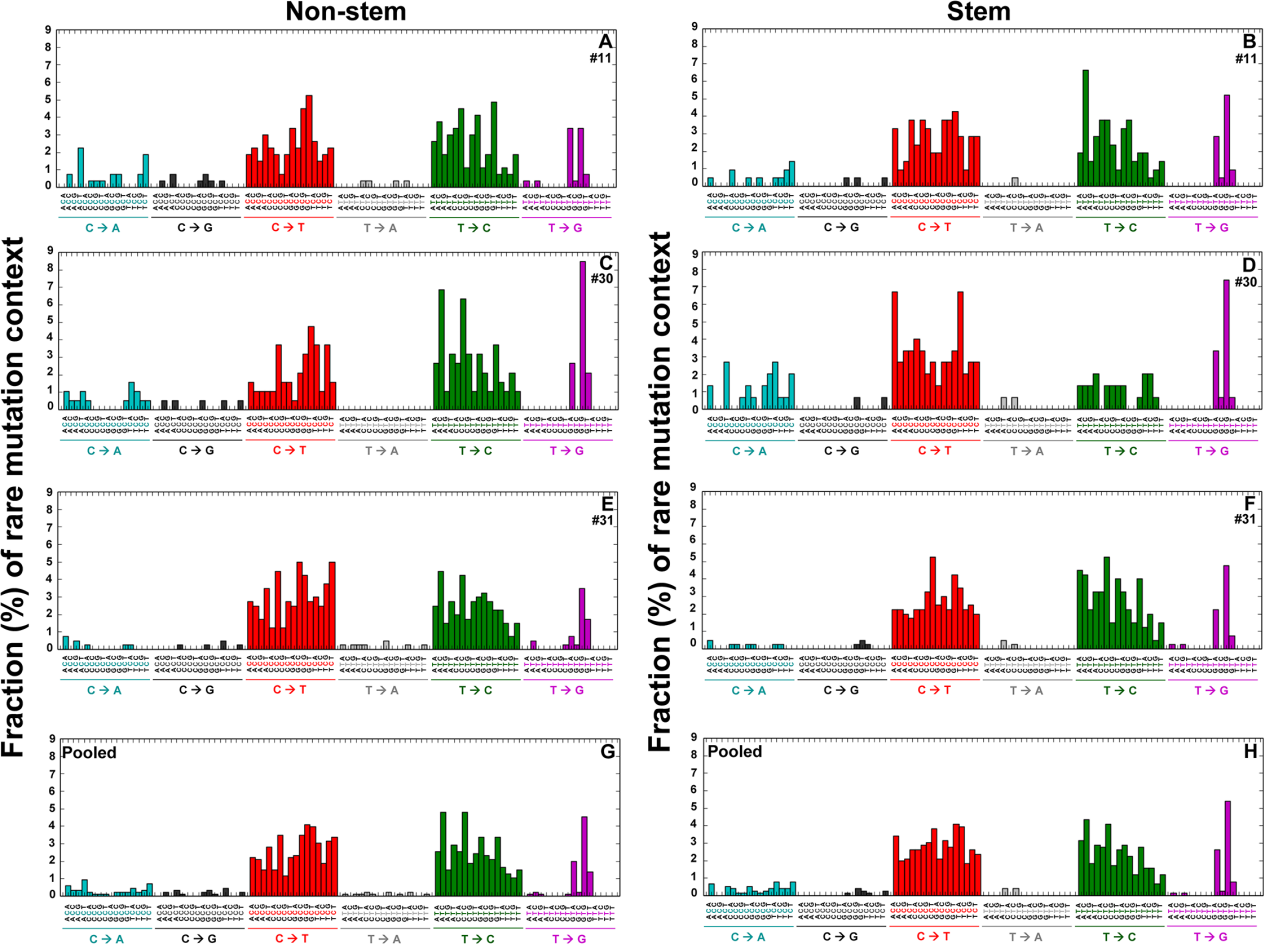
Genome sequence context spectra of rare mutations in the whole mtDNA. Point mutations in the whole mtDNA were determined using DS. The bases immediately 5’ and 3’ to the mutation base (trinucleotides) are calculated as fractions (%) of each type of trinucleotide point mutations (vertical axis) and depict the contribution of each genome sequence context to each point mutation type. The 96 substitution classifications are displayed on the horizontal axes. The graphs list 96 mutation type contexts of one strand, however, the data also represent the complementary mutation context sequences. Data are from human breast normal epithelial cells (non-stem *vs*. stem) developed from women (ID #11, #30, and #31). Pooled data from all three women are shown in (G) and (H).

To investigate whether each rare point mutation type (substitution) occurs in specific genome sequence context and to also investigate how sequence context influences substitution types, the bases immediately 5’ and 3’ to the mutated base (i.e. the mutation occurs at the second position of each trinucleotide) were examined. Fig 4 lists 96 substitution classifications identified. The mutation context for every mutation from each woman is shown in Fig 4A–4F; each sequence context of mutations in normal cells pooled from three women is analyzed (Fig 4G and 4H).

Based on mutation context analysis pooled from three independent sets of cells, 15 types of trinucleotide mutation contexts are observed only in non-stem cells: ACC; ACG for C>A mutations; ACC; ACT; CCA; GCC for C>G mutations; ATA; ATG; CTA; GTA; GTG; TTA; TTT for T>A mutations; and ATC; CTT for T>G mutations (Fig 4G). In comparison, GCA for C>A mutation and GCT for C>G mutation are found only in stem cells (Fig 4H). The two most prevalent types of rare mutations (C>T/G>A and A>G/T>C) are found in 16 types of mutation contexts of both non-stem (Fig 4A, 4C, 4E and 4G) and stem cells (Fig 4B, 4D, 4F and 4H).

Since we observed significantly lower frequencies of rare mutations in stem cells from women (ID# 11 and #30) than in non-stem cells (Fig 1B), we first compared each fraction of mutation contexts for pooled data sets from the two women using the prop.test (‘2-sample test for equality of proportions with continuity correction’). The GTT context for T>C transition (*p* = 0.0234) is significantly higher by 3.2-fold in non-stem cells than in stem cells. The ACA context for C>T (*p* = 0.0259) transition was significantly more prevalent by 2.7-fold in stem cells than in non-stem cells. By comparison, in pooled data from the all three women, the CCG context for C>T transition is significantly higher by 2.6-fold in stem cells than in non-stem cells (*p* = 0.0138) (Fig 4G and 4H). Analyses of point mutation type and sequence context for low-heteroplasmic variants are presented in Supplementary results (S1 Results) and figures (S1 Fig and S2 Fig).

### Homoplasmic variants occur in the similar neighboring bases

To identify homoplasmic point mutations, we used a clonality cutoff that includes genome positions with variants occurring at a frequency of ≥ 95%. We found 54 homoplasmic variants found in any of non-stem or stem cells (S2 Table). Sequence context of homoplasmic point mutations, including the bases immediately 5’ and 3’ to the mutation base are analyzed to examine how sequence contexts affect substitution types (S3 Fig).

Homoplasmic point mutation types are found to be mostly C>T/G>A and A>G/T>C. Furthermore, almost all of the homoplasmic mutations exclusively occur in the same sequence contexts with similar prevalence (Spearman correlation coefficient (*rs*) = 0.96, *p* = 2x10^-7^) in both non-stem and stem cells. Only two homoplasmic mutation sequence contexts (C>T transition in GCG; T>G transversion in GTA) are distinct between non-stem cells and stem cells. Specifically, the GCG for C>T (shown in red) at 5460 mt genome position is only found in non-stem cells developed from a woman (ID #30) (S3A Fig). This C>T transition leads to a nonsynonymous mutation in MT-ND2 coding region, changing alanine to threonine (A331T). In contrast, the T>G transversion (shown in pink), which occurs exclusively in GTA sequence context, is found only in stem cells established from a woman (ID #31) (S3B Fig). This mutation is present in the noncoding region and therefore does not cause changes of proteins.

### Duplex Sequencing identifies known and novel non-homoplasmic variants

We compared all positions of non-homoplasmic variants (rare and low-heteroplasmic variants) to identify common and unique variants between non-stem and stem cells (Tables 1 and 2). We examined whether the common and unique variants we have identified had been already reported by other studies. The gene bank frequency (%) of each identified mutation was calculated based on the previously reported mtDNA variant data base (www.mitomap.org).

**Table 1:** Rare variants identified using Duplex Sequencing in the whole mtDNA of breast normal epithelial cells developed from women (ID#11, #30, #31). Abbreviations used are: Mt, mitochondrial; GB, gene bank; freq, frequency; MERRF, myoclonic epilepsy with ragged red fibers.

Mt gene	DNA variant	Amino acid change	Previously reported (GB freq, %)*	Tissues or diseases reported
**A. Common variants in all sets of non-stem cells from three women**				
RNR1	C1518T	H291Y	*No*	***New variant***
TN	A5705C	T17P	*No*	***New variant***
CO2	G8251A	*None*	Yes (6.36%)	Optic neuropathy, type 2 diabetes, Parkinson's disease, thyroid carcinoma, Alzheimer's disease
ATP8	T8567C	S68P	Yes (0.18%)	Parkinson's disease, breast cancer
ATP6	T8567C	I14T	Yes (0.18%)	Parkinson's disease, breast cancer
ND4L	C10547G	*None*	*No*	***New variant***
ND5	G12684A	*None*	Yes (0.10%)	None
ND5	C12705T	*None*	Yes (39.50%)	Hereditary optic neuropathy, dystonia, respiratory dysfunction, myoclonic epilepsy, MERRF syndrome, Parkinson's disease, schizophrenia, bipolar disorder, major depressive disorder, infantile cardiomyopathy, auditory neuropathy, diabetes, hypertension, Alzheimer's disease, Leigh syndrome
ND5	T13095C	*None*	Yes (0.09%)	Familial sensorineural hearing impairment
ND5	A13105G	I257V	Yes (6.85%)	Antibiotic-induced and non-syndromic deafness, hereditary optic neuropathy
**B. Common variants in all sets of stem cells from three women**				
RNR1	G1476A	V277M	*No*	***New variant***
ATP6	A8577C	*None*	*No*	***New variant***
ND5	G12684A	*None*	Yes (0.10%)	None
ND5	C12705T	*None*	Yes (39.50%)	Hereditary optic neuropathy, dystonia, respiratory dysfunction, myoclonic epilepsy, MERRF syndrome, Parkinson's disease, schizophrenia, bipolar disorder, major depressive disorder, infantile cardiomyopathy, auditory neuropathy, diabetes, hypertension, Alzheimer's disease, Leigh syndrome
ND5	A13062G	*None*	Yes (0.04%)	None
ND5	T13095C	*None*	Yes (0.09%)	Familial sensorineural hearing impairment
ND5	A13105G	I257V	Yes (6.85%)	Antibiotic-induced and non-syndromic deafness, hereditary optic neuropathy
**C. Common variants found in all sets of both non-stem and stem cells from three women**				
ND5	G12684A	*None*	Yes (0.10%)	None
ND5	C12705T	*None*	Yes (39.50%)	Hereditary optic neuropathy, dystonia, respiratory dysfunction, myoclonic epilepsy, MERRF syndrome, Parkinson's disease, schizophrenia, bipolar disorder, major depressive disorder, infantile cardiomyopathy, auditory neuropathy, diabetes, hypertension, Alzheimer's disease, Leigh syndrome
ND5	T13095C	*None*	Yes (0.09%)	Familial sensorineural hearing impairment
ND5	A13105G	I257V	Yes (6.85%)	Antibiotic-induced and non-syndromic deafness, hereditary optic neuropathy
**D. Variants found only in all sets of non-stem, but not in stem cells**				
ATP8	T8567C	S68P	Yes (0.18%)	Parkinson's Disease, breast Cancer
ATP6	T8567C	I14T	Yes (0.18%)	Parkinson's Disease, breast Cancer
ND4L	C10547G	*None*	*No*	***New variant***
**E. Variants found only in all sets of stem cells, but not in non-stem cells**				
None				

* The gene bank (GB) frequency data is derived from 26850 GeneBank sequences with size greater than 14kbp (www.mitomap.org).

**Table 2:** Low-heteroplasmic variants identified using Duplex Sequencing in the whole mtDNA of breast normal epithelial cells developed from women (ID#11, #30, #31).

Mt gene	DNA variant	Amino acid change	Previously reported (GB freq, %)*	Tissues or diseases reported
**A. Common variants in all sets of non-stem cells from three women**				
ND1	A3511C	T69P	*No*	***New variant***
**B. Common variants in all sets of stem cells from three women**				
Non-coding	T310C	*None*	Yes (32.69%)	Parkinson's disease, hereditary optic neuropathy
ND1	A3447C	Q47H	*No*	***New variant***
ATP8	A8512C	K49N	*No*	***New variant***
**C. Common variants found in all sets of both non-stem and stem cells from three women**				
None				
**D. Variants found only in all sets of non-stem, but not in stem cells**				
None				
**E. Variants found only in all sets of stem cells, but not in non-stem cells**				
None				

* The gene bank (GB) frequency data is derived from 26850 GeneBank sequences with size greater than 14kbp (www.mitomap.org).

For rare variants, 10 variants were commonly found in all three sets of non-stem cells from three women (Table 1A) and seven variants were commonly found in all three sets of stem cells (Table 1B). Among these, four variants (m.12684G>A, m.12705C>T, m.13095T>C, m.13105A>G) were found in both sets of non-stem and stem cells from all three women. Interestingly, all of the four common variants were found within the MT-ND5 gene, which has the largest size among the mitochondrial protein-coding genes (Table 1C). The two variants (m.8567T>C; m.10547C>G) in three genes (MT-ATP8; MT-ATP6; MT-ND4L) were found only in all three sets of non-stem cells, but not found in any set of stem cells (Table 1D). Among the common or unique rare variants between non-stem and stem cells, six variants have not been previously reported and we have indicated them as new variants (Table 1 and S3 Table). Among the six variants, three (m.1476G>A, m.1518C>T, m.5705A>C) were nonsynonymous mutations in 12S RNA (MT-RNR1) or the tRNA asparagine (MT-TN) genes.

For low-heteroplasmic variants, m.3511A>C encoding MT-ND1 was the only variant commonly found in all three sets of non-stem cells from three women (Table 2A). The three variants (m.310T>C, m.3447A>C, and m.8512A>C) were found commonly in all three sets of stem cells (Table 2B). No variant was commonly present in both non-stem and stem cells developed from all three women (Table 2C). Among the commonly found low-heteroplasmic variants in non-stem or stem cells that we have identified, three synonymous mutations have not been reported by others (Table 2A and 2B).

### Nonsynonymous mutations occur by chance and the occurrence of nonsynonymous mutations correlates with the size of protein coding genes

Our mutation frequency results indicate that the rare point mutation frequency is lower in stem cells than in non-stem cells (Fig 1B). We further evaluated whether these differences in non-homoplasmic (rare and low-heteroplasmic) mutations can alter protein coding sequences. We quantitated the percentages of nonsynonymous and synonymous mutations within the mutated codons of mtDNA coding regions. Based on the average of pooled data from three independent sets of cells, about 75 to 75.5% of mutations are nonsynonymous (S4 Table). This prevalence of nonsynonymous mutations within the mutated codons is close to the expected value of 75.7% for mtDNA nonsynonymous and synonymous mutations that occur by chance [19]. This suggests that these non-homoplasmic variants are not subject to strong purifying selections and may in fact represent an accumulation of truly random mutations. Consistent with this, the size of each protein-coding gene positively correlates with the prevalence of nonsynonymous mutations in protein coding region (S5 Table: Pearson’s correlation coefficients 0.76–0.95). For instance, nonsynonymous mutations are most prevalent in MT-ND5 gene, of which gene size is the largest among the mt protein encoding genes.

### Effects of nonsynonymous mutations in the mtDNA protein coding regions on the predicted pathogenicity

We evaluated whether non-homoplasmic (rare and low-heteroplasmic) nonsynonymous mutations can alter protein function and increase the predicted pathogenicity using the MutPred program [20]. The MutPred analysis generates *g* scores. A higher *g* score represents a higher probability of an amino acid substitution being deleterious. The sum of *g* scores of codons with nonsynonymous mutations for each gene was pooled for the all of the 13 mitochondrial protein-coding genes. Then the average of the sums of *g* scores for the pooled 13 protein-coding genes was quantitated (S4A Fig). Although the averages of *g* score sums were lower in stem cells than in non-stem cells for women (ID #11 and #30), the differences were not statistically significant. The sum of *g* scores for each gene of each set of normal cells is plotted (S4B–S4D Fig) and described in Supplementary results (S2 Results).

## Discussion

To the best of our knowledge, our current study is the first comprehensive report on mutations of the whole mitochondrial genome in human breast normal epithelial cells (non-stem cells *vs*. stem cells) at the single molecule resolution. Most studies have examined clonal mutations (homoplasmic variants) and have not explored subclonal mutations (heteroplasmic variants) or rare mutations in particular. A major obstacle in investigating rare mutations has been the absence of methods with sufficient sensitivity to accurately detect rare mutations. However, we have established the highly sensitive Duplex Sequencing (DS) method that enables the genome-wide measurement of non-homoplasmic (rare and heteroplasmic) mutation frequency, spectrum, and distribution. Our DS method presents the greatest accuracy (error rates 5x10^-8^ to 10^−8^) among currently available high-throughput sequencing methods such as routine NGS (error rate 10^−2^ to 10^−3^) [7,9–11].

The frequency of rare mutations is proportional to both the rate of *de novo* mutations per generation and the total number of cell divisions happened during development and aging [21]. Here we demonstrate that, in two of three paired normal epithelial cells, the overall rare mutation frequencies of the whole mtDNA are significantly lower in stem cells than in non-stem cells (Fig 1B). A possible mechanism of this lower rare mutation frequency in stem cells might be associated with reduced production of reactive oxygen species (ROS). The lower ROS levels in stem cells could lead to reduced mtDNA damage and reduced accumulation of rare mutations. In human hematopoietic stem cells, lower ROS levels were required to maintain stem cell features [22,23], while increased ROS levels initiated differentiation by activating p38 mitogen activated protein kinase (MAPK). Both murine and human embryonic stem cell cultures produced fewer ROS than most somatic cell types as a result of less oxidative phosphorylation and lower mitochondrial biogenesis [24,25]. Alternatively, stem cells can have more efficient systems of DNA repair and mitophagy in removing damaged mtDNA [26,27,28], thereby lowering mtDNA mutations. Another possibility is that mitochondria with damaged DNA simply do not replicate and are not passed on as cells differentiate.

Our mutation spectrum analyses indicate that the C>T/G>A and A>G/T>C transitions are the two most prevalent substitution types of mtDNA in rare (Figs 2–4), low-heteroplasmic (S1 and S2 Figs) and homoplasmic (S3 Fig) mutations. It was reported that transitions are more frequent than transversions in the mtDNA of humans [29,30] and in 70 species of animals [31]. The C>T/G>A mutation is possibly due to misincorporation by DNA polymerase γ or the deamination of cytosine to form uracil [32]. These have been reported to be major drivers of mtDNA mutation [33,34]. A>G/T>C mutation could be due to the deamination of adenine to inosine or T-dGTP mispairing: a major base misinsertion made by DNA polymerase γ [35–38]. In addition, C>T/G>A and A>G/T>C transitions can positively correlate with ROS levels [39,40].

A large amount of DNA damage is generated by oxygen free radicals. Among these, 8-oxo-2’-deoxyguanosine (8-oxo-dG), produced by the oxidation of DNA under physiopathological conditions or environmental stress or by a by-product of normal cellular metabolism [41,42,43], is among the most thoroughly investigated [44,45]. The 8-oxo-dG has been shown to preferentially pair with dA [46] resulting in G>T (/C>A) transversions. It is believed that cells have evolved a number of enzymatic mechanisms that prevent incorporation of 8-oxo-dG into DNA, by excising 8-oxo-dG and dA, the complement to 8-oxo-dG in DNA. We have found that the C>A/G>T transversion only accounts for 6 to 8% of the overall rare mutations (Fig 3B). A recent DS study with human brain tissues also found that only a small fraction of C>A/G>T makes up mtDNA rare mutations [8]. Thus, this low frequency of C>A/G>T mutations in the whole mtDNA of human breast normal cells and brain tissues may suggest three possible scenarios: 8-oxo-dG is barely produced in these cells or tissues, mtDNA 8-oxo-dG lesions are rapidly repaired, or the damaged mtDNA is quickly degraded through mitophagy.

We have detected a strand bias of mtDNA in human breast normal epithelial cells (Fig 2A–2F). G>A is more prevalent than C>T; T>C is more prevalent than A>G; A>C is more prevalent than T>G on the light strand of the mitochondrial genome. These asymmetric distributions reflect a strand bias, a phenomenon which has previously been reported in human population studies [31,47,48] and in mtDNA mutations of human brain [8], human putamen [49], fruit fly *Drosophila melanogaster* [50], and human tumors [51,52]. Origins for strand biases have been suggested to arise during mtDNA asymmetric replication [53,54]. An excess of G>A transitions was demonstrated *in vitro* in small regions of mtDNA amplified by polymerase γ and PCR [38].

Our mutation context analysis has highlighted differences between non-stem cells and stem cells in the sequence context of non-homoplasmic (rare and low-heteroplasmic) mutations (Fig 4 and S2 Fig). In pooled data sets from three women, 15 types of rare mutation context are observed in non-stem cells, but not in stem cells. The CCG context for C>T transition is significantly higher in stem cells than in non-stem cells (*p* = 0.0138, Pooled data: Fig 4G–4H). Given the apparent absence of purifying selection, these results suggest that distinct mutational processes may be operative in breast non-stem cells and stem cells. Dissimilarities identified by 96 mutation context classifications could aid in explaining deviations in phenotypes, sensitivities to transformation and to potential chemotherapeutic drugs between human breast normal non-stem cells and stem cells [13–15]. In contrast, the sequence contexts of homoplasmic mutations (S3 Fig) are almost identical (Correlation coefficient = 0.96) between non-stem cells and stem cells, confirming that both cell types are derived from the corresponding same woman.

We have identified common non-homoplasmic variants present in both non-stem and stem cells from all three women and have also identified unique non-homoplasmic variants found only in non-stem or stem cells. We have found that only four non-homoplasmic variants are commonly present in all three women (Tables 1 and 2), which suggests a substantial inter-personal variation of the mitochondrial genome. A recent study demonstrated extensive diversity in point mutations of nuclear DNA occurring at low frequencies (<10%) among breast tumors from different patients and within the same patient’s breast tumors [55]. Among the common or unique non-homoplasmic variants identified in our study, we have found nine novel non-homoplasmic variants (S3 Table) that have not been reported by others. In contrast, all homoplasmic variants found in our study were reported previously (S2 Table).

Our data indicate that nonsynonymous and synonymous mutations occur as would be expected based on stochastic events in the absence of selection. Consistent with this, the size of each mitochondrial protein-coding gene positively and strongly correlates with the prevalence of nonsynonymous mutations in the mtDNA protein coding region (S5 Table: Pearson’s correlation coefficients 0.76−0.95). A positive correlation between the mtDNA protein coding gene size and number of nonsynonymous mutations was also observed in breast tumor and matched normal tissue data obtained using conventional NGS [51].

In summary, by using the ultra-sensitive Duplex Sequencing method, we have found that the majority of mutational variation occurs at the random level. Some of the variants that we detected are new variants, which have not been reported previously. Our data indicate that the mitochondrial genomes of stem cells carry lower rare mutation burdens than those of non-stem cells and that the most prevalent point mutation types are the C>T/G>A and A>G/T>C transitions. In addition, substantial variations exist in mitochondrial non-homoplasmic variants between individuals. Our results of human mitochondrial mutation signatures will aid in characterizing normal breast epithelial cells and may form a foundation to characterize cancer stem cell mutation profiles.

## Materials and Methods

### Development and culture of human breast normal epithelial cells

Breast tissues of healthy women at 21–29 years of age were obtained during reduction mammoplasty at Sparrow Hospital (SH) in Lansing, MI and patients’ written consents were obtained in accordance with institutional guidelines. Normal breast cells were isolated from breast tissues at Michigan State University (MSU). All of these procedures were approved by SH Institutional Review Board and MSU Human Research Projection Program to conduct this study. MSU Technologies and the University of Washington Center for Commercialization approved the use of the cells for this research and it was documented in Material Transfer Agreement. In contrast to human breast normal non-stem cells, normal stem cells have been previously characterized by the deficiency in gap-junctional intercellular communication [12], the ability of anchorage-independent growth [13], the ability to differentiate into basal and luminal epithelial cells [12,13], reduced expression of maspin [15], the expression of estrogen receptor-alpha [56] and a stem cell marker OCT4 [14]. These two types of cells were separated and isolated based on their differences in cell attachment on a surface, growth response to fetal bovine serum (FBS), bovine pituitary extract (BPE) (Invitrogen, Grand Island, NY), and cell morphology [12, 15].

Normal cells were cultured for up to, on average, 24 days after thawing the frozen primary cells isolated from breast tissues and maintained as previously described [12,15]. Stem cells were cultured in MSU-1+S medium with FBS, while non-stem cells were cultured in MSU-1+S medium with BPE [15]. The normal cells passaged up to three times or less were used for experiments. The normal cells used in this study are designated as women ID# 11, #30, and #31.

### DNA extraction and mitochondrial (mt) DNA copy number quantitation

Total DNA was isolated with a lysis buffer (10 mM Tris-HCl, pH 8.0, 150 mM NaCl, 20 mM EDTA, 1% SDS) and a commercially available DNA extraction kit (Invitrogen, Grand Island, NY). The mtDNA content per haploid genome cell was determined by QPCR in two separate reactions: one with a primer pair complementary to mitochondrial DNA (forward: ACAGTT TATGTA GCTTAC CTCC and reverse: TTGCTG CGTGCT TGATGC TTGT) to quantitate mitochondrial genomes, and one with a primer pair complementary to the single copy nuclear gene in a region of chromosome 17:8465423–8465538 (forward: TTGCCA GACCAT GGGATT GTCTCA and reverse: TTCCTA CCGAAC GAGGAC TCCAAA) to quantitate nuclear genomes. PCR reactions were carried out in triplicates of DNA in 25 μL reaction volumes with Brilliant III Ultra-Fast SYBR Green QPCR Master Mix (*cat#* 600882, Agilent Technologies) and 10 μM forward and 10 μM reverse primers. Alternatively, Brilliant SYBR Green QPCR Master Mix (cat# 600548, Stratagene) with 1 unit UDG (New Englad Biolab) was used instead of Brilliant III Ultra-Fast SYBR Green QPCR Master Mix.

By average, 425 to 479 copies of mtDNA per haploid genome were found in normal non-stem and stem cells. No significant difference in mtDNA copy number was observed between the two cell types (S5 Fig).

### Adapter synthesis and DNA library preparation for Duplex Sequencing

Duplex Tag-labeled adapters with a single-nucleotide A [7] or T overhang [9] were synthesized as previously described. Sequencing library preparation was carried out as previously described [7,9] with modifications. For adapters with A-overhang, the sheared DNA was subjected to end-repair and 3’-dT-tailing. For adapters with T-overhang, the sheared DNA was processed to end-repair and 3’-dA-tailing. Prior to the ligation of DNA with the adapters, DNA was quantitated using QPCR and the ratio of mtDNA relative to nuclear DNA was determined. After the ligation by adding 20-fold molar excess of adapter relative to DNA and purification, DNA was amplified and the whole mitochondrial genome was captured using Agilent SureSelect^XT^ target enrichment (Agilent Technologies, Santa Clara, CA). DNA libraries were sequenced on Illumina HiSeq 2500 platform (Illumina Inc., San Diego, CA).

### Duplex Sequencing (DS) data processing and analysis

DS data were processed as described [7,9] with minor modifications. The reads obtained from DS runs were aligned to the reference genome using BWA version 0.6.2–1 [57]. Revised Cambridge Reference sequence (rCRS), available as sequence number NC_012920 (formerly AC_000021.2) in Genebank’s RefSeq database, was used as the reference for the whole mtDNA genome. In order to eliminate potential artifactual mutations arising from the end-repair and ligation reactions, each read was subjected to local realignment with genome analysis toolkit (GATK) version 2.3.9-ge5ebf34 [58] and was followed by clipping of the first five bases at 5’ ends and of the last six bases at 3’ ends of each read. Mutation signatures and amino acid changes were analyzed using in-house written scripts. The latest version of the DS software package and scripts developed by our group can be downloaded from https://github.com/loeblab/Duplex-Sequencing. Scripts for mutation context signatures and amino acid changes (nonsynonymous and synonymous mutations) are described in Supplementary Methods (S1 Methods). The overall frequencies of rare mutations are calculated as the total number of mutant nucleotides divided by the total number of sequenced nucleotides and the mutants are scored only once at each position of the genome.

### Pathogenicity of nonsynonymous mutations

The prediction of pathogenicity for nonsynonymous mutations in the mitochondrial protein-coding genes was analyzed using the MutPred web application tool version 1.20 (http://mutpred.mutdb.org). The MutPred analysis generates general (*g*) scores. The *g* score, ranging 0 to 1, is assigned to each nonsynonymous mutation and represents the probability that the amino acid substitution is deleterious or disease-associated [20].

### Statistical analysis

Differences in mtDNA mutation frequencies and mutation counts between the two groups (non-stem *vs*. stem) were analyzed by performing the prop.test for ‘2-sample test for equality of proportions with continuity correction’ using R program version 3.0.2. The Mann-Whitney U-test (Wilcoxon rank-sum test) was applied to compare *g* scores of MutPred data and fractions of mutation types between the two groups. Associations for the mutation context signatures of homoplasmic mutations between the two groups were examined by Spearman correlation coefficients (*rs*). The associations of protein coding gene sizes with numbers of nonsynonymous mutations were evaluated by Pearson’s correlation coefficients (*r*). Unless it is noted, Sigma Plot version 12.0 program was used for statistical analyses. Differences between the two groups were considered significant when the *p* value was less than 0.05.

## Supporting Information

S1 Fig Types of low-heteroplasmic mutations in the whole mtDNA. Types of low-heteroplasmic point mutations and insertions and deletions (INDELs) in the whole mtDNA were determined using DS. Data are from human breast normal epithelial cells (non-stem *vs*. stem) developed from women (ID #11, #30, and #31). Error bars represent the Wilson Score 95% confidence intervals.
(TIF)

S2 Fig Genome sequence context spectra of low-heteroplasmic mutations in the whole mtDNA. Point mutations of the whole mtDNA were determined using DS. The bases immediately 5’ and 3’ to the mutation base (trinucleotides) are calculated as fractions (%) of each type of trinucleotide point mutation (vertical axis) and depict the contribution of each genome sequence context to each point mutation type. The 96 substitution classifications are displayed on the horizontal axes. The graphs list 96 mutation type contexts of one strand; however, the data also represent the complementary mutation context sequences. Data are from human breast normal epithelial cells (non-stem *vs*. stem) developed from women (ID #11, #30, and #31). Pooled data from all three women are shown in (G) and (H).
(TIF)

S3 Fig Genome sequence context spectra of homoplasmic mutations in the whole mtDNA. Point mutations of the whole mtDNA were determined using DS. The bases immediately 5’ and 3’ to the mutation base (trinucleotides) are calculated as fractions (%) of each type of trinucleotide point mutation (vertical axis) and depict the contribution of each genome sequence context to each point mutation type. The 96 substitution classifications are displayed on the horizontal axes. The graphs list 96 mutation type contexts of one strand; however, the data also represent the complementary mutation context sequences. Data are pooled from human breast normal epithelial cells (non-stem *vs*. stem) developed from all three women.
(TIF)

S4 Fig Effects of non-homoplasmic nonsynonymous mutations of the mtDNA protein coding regions on the predicted pathogenicity. The *g* scores of non-homoplasmic (rare and low-heteroplasmic) point mutations within the mtDNA protein coding sequences were obtained from MutPred web-based analysis. Data are from human breast normal epithelial cells (non-stem *vs*. stem) developed from women (ID #11, #30, and #31).
(TIF)

S5 Fig Copy numbers of mtDNA per haploid nuclear genome. The mtDNA copy numbers were quantitated using QPCR. Two independent culture experiments for the paired normal cells from two women (ID #30 and #31) are shown (Mean ± S.E.M.).
(TIF)

S1 Methods (a) Script for mutation context signature analysis. (b) Script for amino acid changes (Note: Do not open this script file using a word program. It will change formats of commands and the commands will not work). (TXT)

S1 Results Mutation signatures of low-heteroplasmic mutations in the whole mtDNA. (DOCX)

S2 Results Predicted pathogenicity scores for nonsynonymous mutations of non-homoplasmic variants in mitochondrial protein coding genes. (DOCX)

S1 Table Data yield and Duplex Sequencing statistics. (DOCX)

S2 Table Homoplasmic variants found in any of non-stem or stem cells developed from women (ID #11, #30, and #31). (DOCX)

S3 Table New non-homoplasmic variants within the common and unique mutations identified between non-stem and stem cells. (DOCX)

S4 Table Distribution of nonsynonymous mutations of non-homoplasmic variants within mtDNA coding regions. (DOCX)

S5 Table The number of non-synonymous mutations of non-homoplasmic variants in mitochondrial protein coding genes. (DOCX)
