# Supplementary figures and images for: Detection of Ultra-Rare Mitochondrial Mutations in Breast Stem Cells by Duplex Sequencing
Source: PLoS One. 2015 Aug 25;10(8):e0136216. doi: 10.1371/journal.pone.0136216 (PMC4549069; doi:10.1371/journal.pone.0136216)

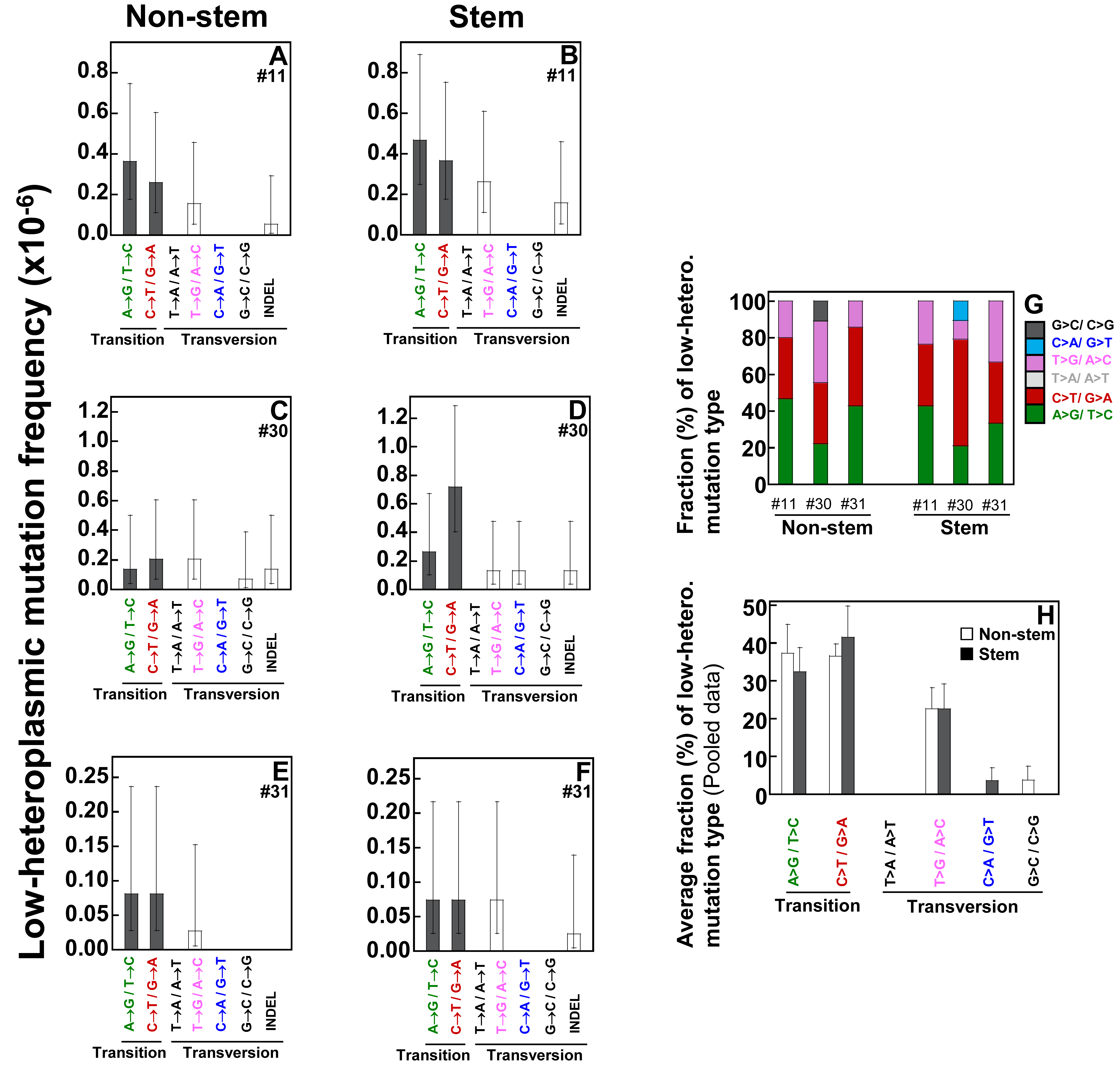

Supplement: S1 Fig — Types of low-heteroplasmic point mutations and insertions and deletions (INDELs) in the whole mtDNA were determined using DS. Data are from human breast normal epithelial cells (non-stem vs. stem) developed from women (ID #11, #30, and #31). Error bars represent the Wilson Score 95% confidence intervals. (TIF) [file pone.0136216.s001.tif]

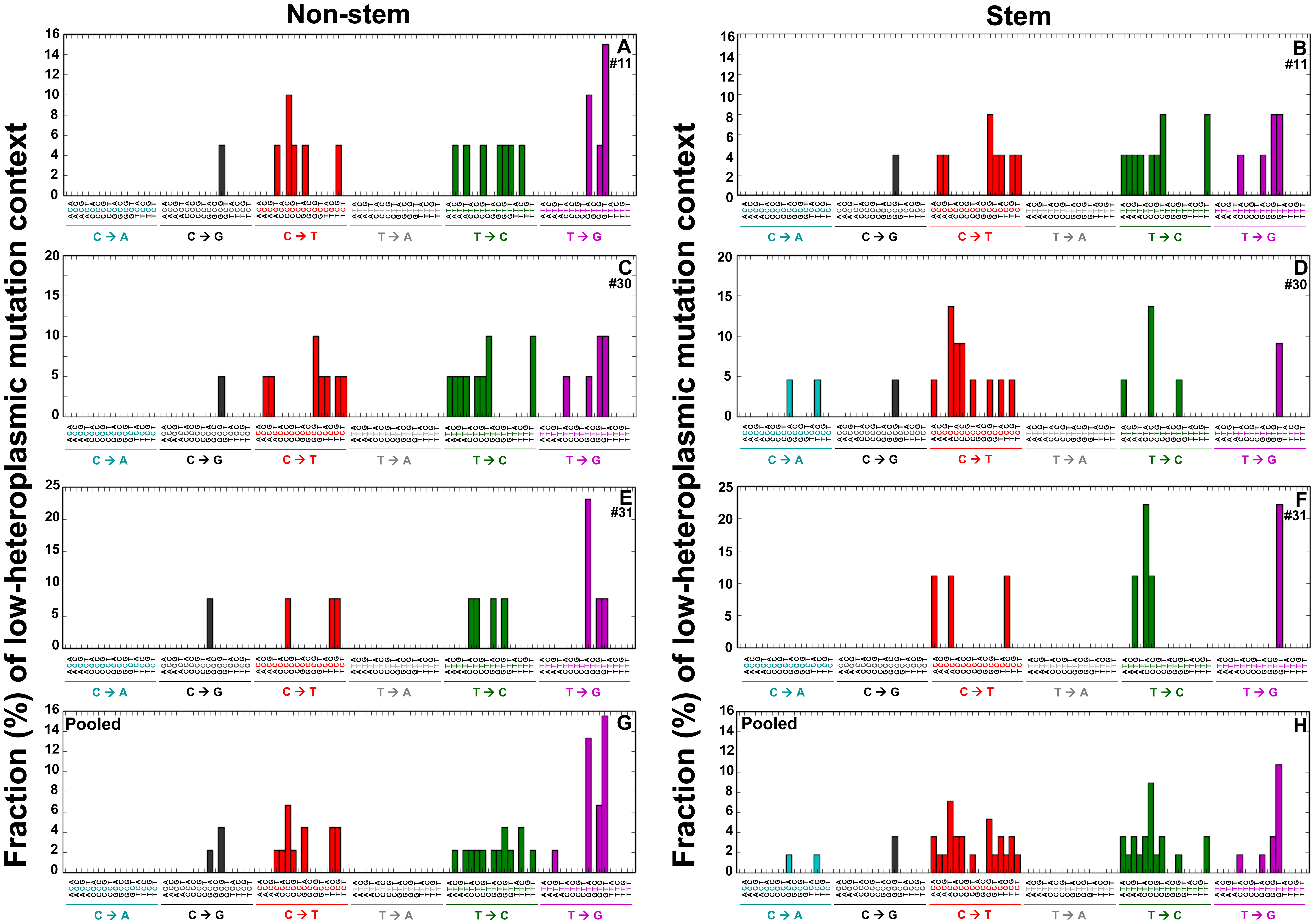

Supplement: S2 Fig — Point mutations of the whole mtDNA were determined using DS. The bases immediately 5’ and 3’ to the mutation base (trinucleotides) are calculated as fractions (%) of each type of trinucleotide point mutation (vertical axis) and depict the contribution of each genome sequence context to each point mutation type. The 96 substitution classifications are displayed on the horizontal axes. The graphs list 96 mutation type contexts of one strand; however, the data also represent the complementary mutation context sequences. Data are from human breast normal epithelial cells (non-stem vs. stem) developed from women (ID #11, #30, and #31). Pooled data from all three women are shown in (G) and (H). (TIF) [file pone.0136216.s002.tif]

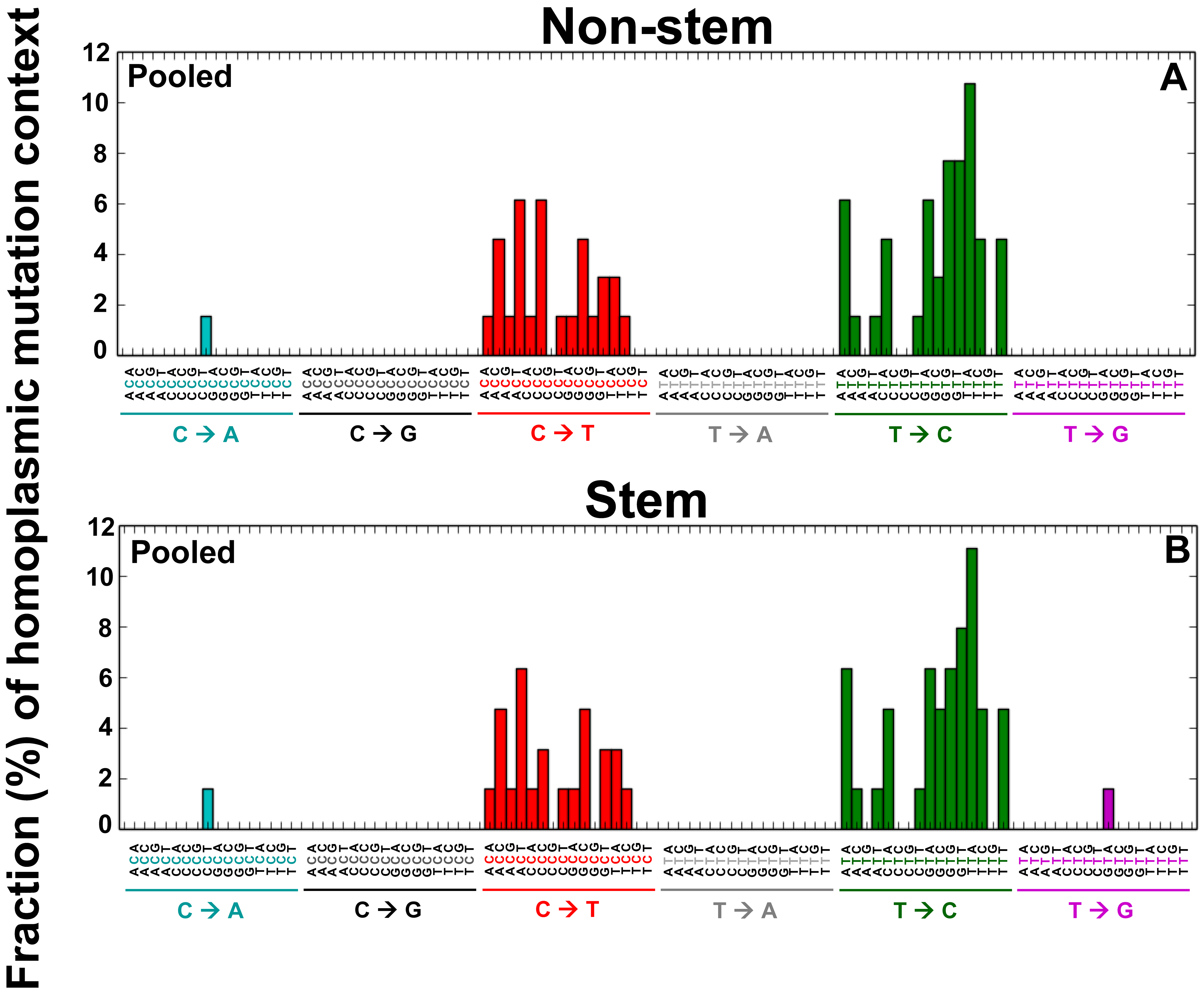

Supplement: S3 Fig — Point mutations of the whole mtDNA were determined using DS. The bases immediately 5’ and 3’ to the mutation base (trinucleotides) are calculated as fractions (%) of each type of trinucleotide point mutation (vertical axis) and depict the contribution of each genome sequence context to each point mutation type. The 96 substitution classifications are displayed on the horizontal axes. The graphs list 96 mutation type contexts of one strand; however, the data also represent the complementary mutation context sequences. Data are pooled from human breast normal epithelial cells (non-stem vs. stem) developed from all three women. (TIF) [file pone.0136216.s003.tif]

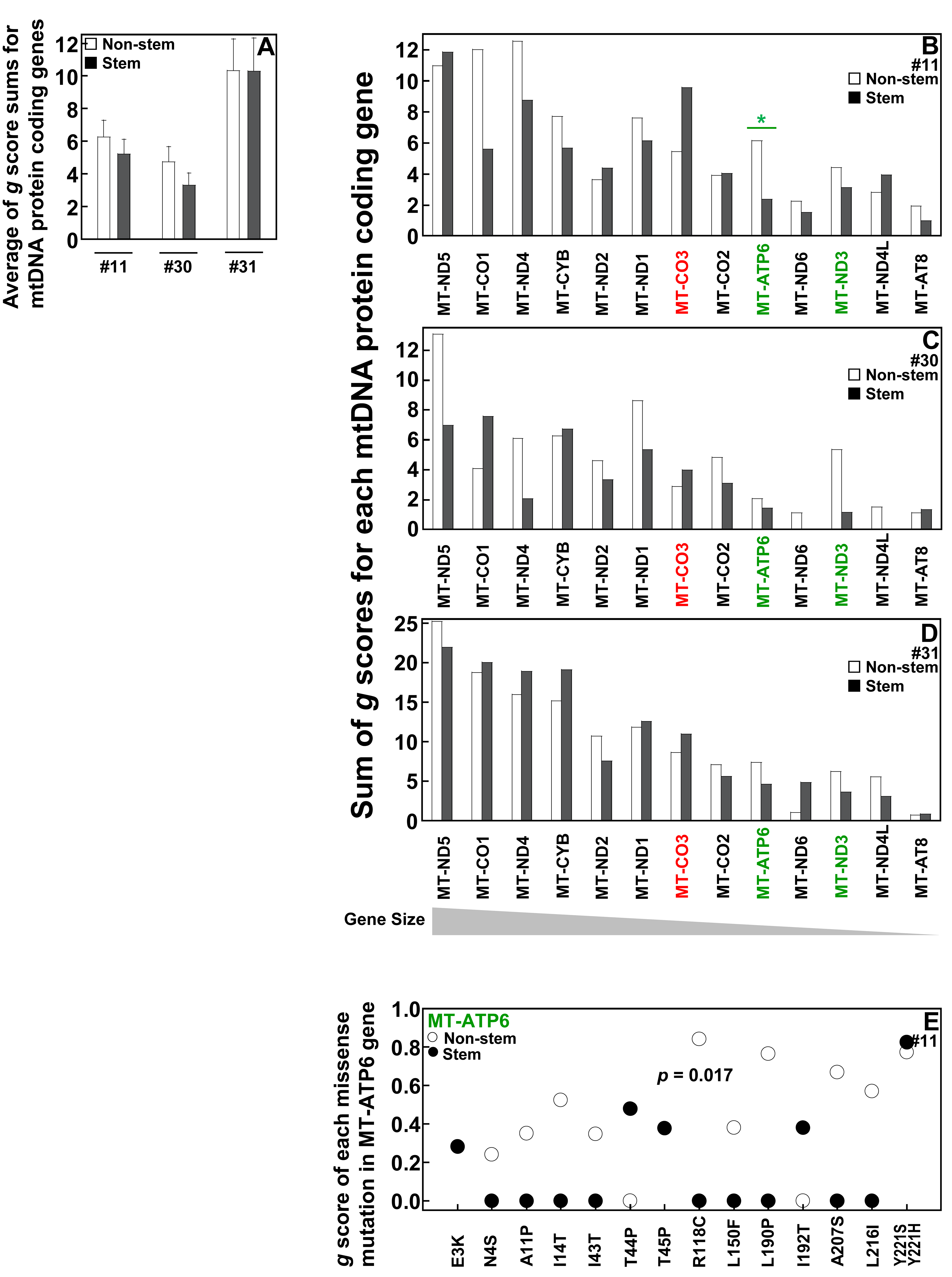

Supplement: S4 Fig — The g scores of non-homoplasmic (rare and low-heteroplasmic) point mutations within the mtDNA protein coding sequences were obtained from MutPred web-based analysis. Data are from human breast normal epithelial cells (non-stem vs. stem) developed from women (ID #11, #30, and #31). (TIF) [file pone.0136216.s004.tif]

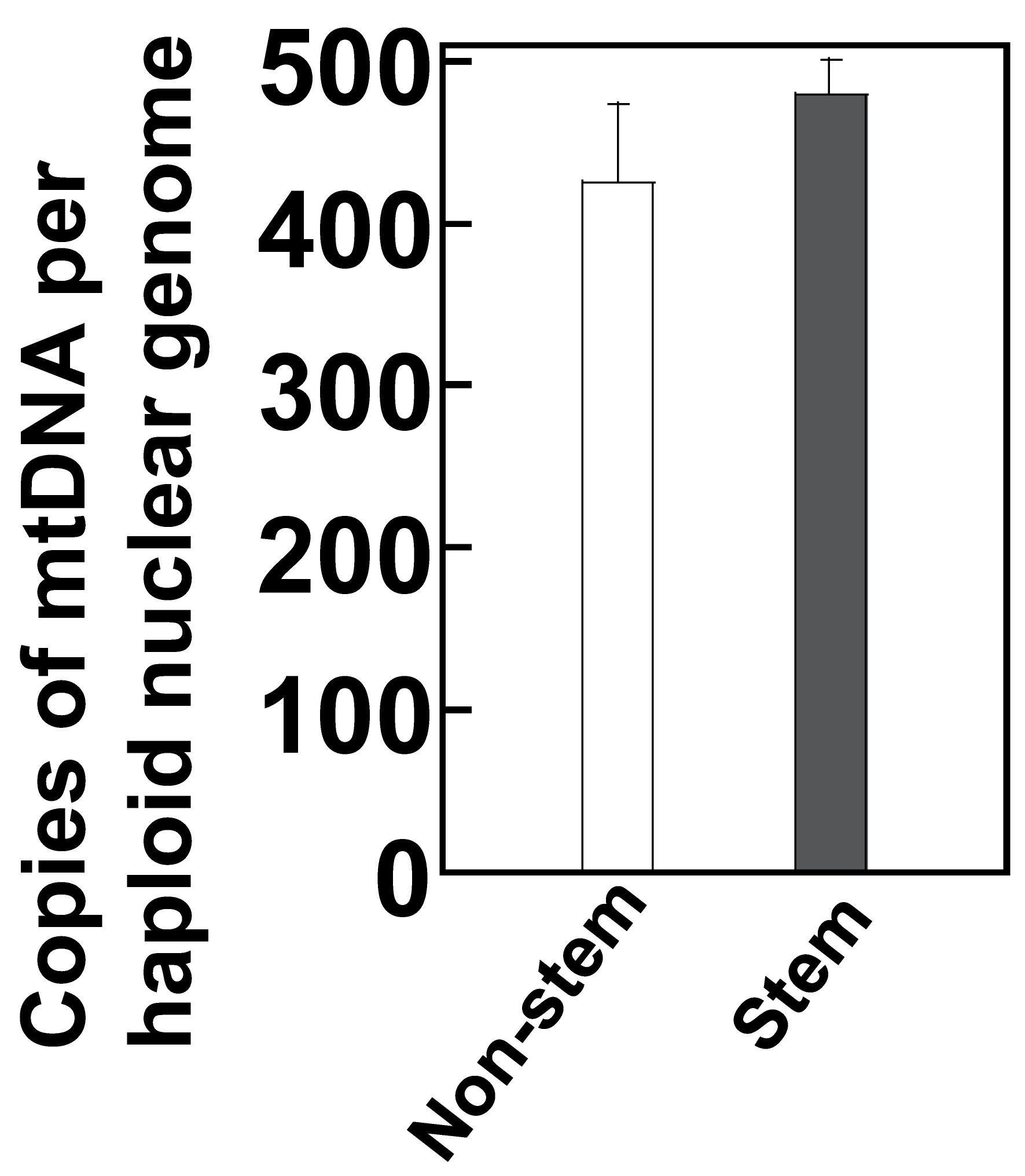

Supplement: S5 Fig — The mtDNA copy numbers were quantitated using QPCR. Two independent culture experiments for the paired normal cells from two women (ID #30 and #31) are shown (Mean ± S.E.M.). (TIF) [file pone.0136216.s005.tif]
