# Supplementary material for: Detection of Ultra-Rare Mitochondrial Mutations in Breast Stem Cells by Duplex Sequencing
Source: PLoS One. 2015 Aug 25;10(8):e0136216. doi: 10.1371/journal.pone.0136216 (PMC4549069; doi:10.1371/journal.pone.0136216)
Supplement: S1 Results — (DOCX) [file pone.0136216.s007.docx]

**Supplementary Results: S1 Results**

**Mutation signatures of low-heteroplasmic mutations in the whole mtDNA**

**Types of low-heteroplasmic mutations in the whole mtDNA.**

We have examined the types of point mutations and INDELs that occur at low-heteroplasmic clonality ranges (>0.5 to 20%). Since low-heteroplasmic mutations are only small fractions of all mutations (Fig. 1A), 12 substitution types are consolidated into six mutation types. The C>T/G>A and A>G/T>C transitions are still the most prevalent random mutation types, followed by the T>G/A>C transversion (S2 Fig.). The T>A/A>T transversion is found neither in non-stem nor in stem cells. G>C/C>G transversion is only found in non-stem cells from woman ID# 30 (S2C, G Figs.), while C>A/G>T is only found in stem cells from woman ID# 30 (S2D, G Figs.). No INDEL is found in non-stem cells from woman ID# 31 (S2E Fig.).

Each type of mutation is presented as a mutant fraction (%) of overall low-heteroplasmic mutations for each primary cell model isolated from an each woman (S2G Fig.). For primary normal cells isolated from a woman (ID# 30), stem cells have a higher fraction (58%) of the C>T/G>A (shown in red) transition than do non-stem cells (33%), but stem cells have a lower fraction (11%) of T>G/A>C than do non-stem cells (33%). Only a minor difference between non-stem and stem cells is observed in other primary cells established from women ID# 11 and #31 in fractions (%) of each substitution type. The average fractions (%) of each mutation type of primary cells pooled from three women are also quantitated (S2H Fig.). The pooled average fraction results indicate that the relative occurrence of each mutation type is not significantly different between non-stem and stem cells.

**Neighboring bases influence the frequencies and types of low-heteroplasmic mutations.**

The bases immediately 5’ and 3’ to the mutation base (i.e. the mutation is on the second base of trinucleotides) are analyzed to examine how sequence context affects substitution types (S3 Fig.). The graphs shown in S3 Figure list 96 substitution classifications of one strand; however, the data also represent the complementary sequence contexts. The mutation context of each normal cell model from each woman is shown in S3A-F Figs. Furthermore, each sequence context of mutations in normal cells pooled from three women is analyzed (S3G, H Figs.).

Based on the pooled data of low-heteroplasmic mutation context analysis, eight types of trinucleotide mutation context types are found only in non-stem cells: GCA for C>G mutations; CCG, GCA for C>T; GTA, GTC, GTT, TTC for T>C; ATG for T>C mutation (S3G Fig.**)**. In comparison, 14 types of mutation contexts are only observed in stem cells: GCA, TCC for C>A mutations; ACA, ACC, ACG, CCT, GCG, GCT, TCA, TCT for C>T; ATA, ATG, CTT for T>C; CTA for T>G (S3H Fig.). Some sequence contexts are more frequently found in stem cells than in non-stem cells, or vice versa. For example, the CTC fraction for T>C transition is about 3.5 fold higher in stem cells than in non-stem cells. The GTA fraction for the T>G transversion is about 6.5-fold higher in non-stem cells than in stem cells.
