# Supplementary material for: Detection of Ultra-Rare Mitochondrial Mutations in Breast Stem Cells by Duplex Sequencing
Source: PLoS One. 2015 Aug 25;10(8):e0136216. doi: 10.1371/journal.pone.0136216 (PMC4549069; doi:10.1371/journal.pone.0136216)
Supplement: S2 Results — (DOCX) [file pone.0136216.s008.docx]

**Supplementary Results: S2 Results**

**Predicted pathogenicity scores for nonsynonymous mutations of non-homoplasmic variants in mitochondrial protein coding genes**

The sum of *g* scores for each gene of each set of normal cells is plotted (S5B-D Figs.). Although variations among the women existed, three genes consistently showed similar trends in all women. For example, the sums of *g* scores for MT-CO3 (highlighted in red) were consistently higher in all three independent stem cells, than in non-stem cells (S5B-D Figs.). The sums of *g* scores for MT-ATP6 and MT-ND3 (highlighted in green) were consistently higher in all three independent non-stem cells than in stem cells (S5B-D Figs.). Thus, we further examined each *g* score for each nonsynonymous mutation of these three genes (MT-CO3, MT-ATP6, and MT-ND3), in each set of stem cells and non-stem cells (S5B-D Figs.). Among these three genes, the *g* scores for MT-ATP6 were significantly higher in non-stem cells from one of the women, ID #11, than those of the corresponding stem cells (S5E Fig.: p=0.017 by Mann-Whitney U test); other differences were not statistically significant. Within the women (ID #11 and #30), there were three other genes (MT-ND1, MT-ND4, and MT-ND6) for which the *g* score sums were consistently higher in non-stem cells than in the corresponding stem cells (S5B,C Figs.).

In summary, although the averages of the *g* score sums for mt protein-coding genes were not significantly different between non-stem cells and stem cells, tendencies for lower averages of the *g* score sums were observed in stem cells than in non-stem cells. This is consistent with the idea that stem cell mtDNA can be protected from deleterious mutation accumulation.
