# Supplementary material for: Detection of Ultra-Rare Mitochondrial Mutations in Breast Stem Cells by Duplex Sequencing
Source: PLoS One. 2015 Aug 25;10(8):e0136216. doi: 10.1371/journal.pone.0136216 (PMC4549069; doi:10.1371/journal.pone.0136216)
Supplement: S1 Table — (DOCX) [file pone.0136216.s009.docx]

**S1 Table.** Data yield and duplex sequencing statistics.

| Sample ID | Avg SSCS depth | Total sequenced SSCS Nts | SSCS:  No. of rare muts | **SSCS: Rare mut freq** | Avg DCS depth | Total sequenced DCS Nts | % reads mapping to mt | DCS:  No. of rare muts | **DCS: Rare mut freq** | DCS:  No. of low-heteroplasmic muts | DCS: Low-heteroplasmic mut freq |
| --- | --- | --- | --- | --- | --- | --- | --- | --- | --- | --- | --- |
| **HME 11**  Non-stem | 4488 | 74358904 | 4287 | 5.77x10^-5^ | 1170 | 19389999 | 65 | 262 | 1.35x10^-5^ | 15 | 7.74x10^-7^ |
| Stem | 7219 | 119606415 | 5244 | 4.38 x10^-5^ | 1163 | 19262440 | 75 | 207 | 1.07x10^-5^ | 21 | 1.09x10^-6^ |
| **HME 30**  Non-stem | 3272 | 54205589 | 4562 | 8.42 x10^-5^ | 883 | 14625056 | 66 | 176 | 1.20x10^-5^ | 9 | 6.15x10^-7^ |
| Stem | 3338 | 55303748 | 3054 | 5.52 x10^-5^ | 924 | 15316422 | 69 | 145 | 9.47x10^-6^ | 19 | 1.24x10^-6^ |
| **HME 31**  Non-stem | 12256 | 203075124 | 5364 | 2.64 x10^-5^ | 2248 | 37240333 | 88 | 397 | 1.07x10^-5^ | 7 | 1.88x10^-7^ |
| Stem | 11045 | 183005098 | 7416 | 4.05 x10^-5^ | 2460 | 40754403 | 61 | 395 | 9.69x10^-6^ | 9 | 2.21x10^-7^ |

Abbreviations used are: Avg, average; Nts, nucleotides; SSCS, single strand consensus sequences; DCS, duplex consensus sequences; mt, mitochondria; mut, mutation; freq, frequency.
