# Supplementary material for: Detection of Ultra-Rare Mitochondrial Mutations in Breast Stem Cells by Duplex Sequencing
Source: PLoS One. 2015 Aug 25;10(8):e0136216. doi: 10.1371/journal.pone.0136216 (PMC4549069; doi:10.1371/journal.pone.0136216)
Supplement: S2 Table — (DOCX) [file pone.0136216.s010.docx]

**S2 Table.** Homoplasmic variants found in any of non-stem or stem cells developed from three women (ID #11, #30, and #31). In total, 54 variants are identified and all variants have previously been identified.

| Mt gene | DNA variant | Amino acid change | GB freq(%)* | Tissues or diseases reported** |
| --- | --- | --- | --- | --- |
| Control region | A73G | Non-coding | 72.62% | Aging brains, POLG/PEO & control muscle, buccal cell, thyroid & prostate tumors |
| Control region | T152C | Non-coding | 22.87% | Aging brains, elderly fibroblasts, ovarian carcinoma, breast tumor |
| Control region | G207A | Non-coding | 4.55% | Oral, prostate and thyroid tumor |
| Control region | A263G | Non-coding | 92.86% | POLG and MNGIE muscle |
| MT-RNR1 | G709A | R21H | 13.10% |  |
| MT-RNR1 | A750G | N35D | 98.54% |  |
| MT-RNR1 | G930A | A95T | 2.13% |  |
| MT-RNR1 | A1438G | Ter264W | 94.48% |  |
| MT-RNR2 | T1700C | *none* | 0.74% |  |
| MT-RNR2 | G1888A | S73N | 5.74% |  |
| MT-RNR2 | A2706G | N346D | 76.04% |  |
| MT-RNR2 | C2772T | H368Y | 0.47% |  |
| MT-RNR2 | T3197C | V509A | 4.38% |  |
| MT-ND1 | T3398C | M31T | 0.35% | DMDF +HCM / GDM / possibly LVNC cardiomyopathy-associated |
| MT-ND1 | T4216C | Y304H | 10.34% | Acute leukemia platelets, leukocytes and bone marrow |
| MT-ND2 | A4769G | *none* | 97.69% |  |
| MT-ND2 | A4917G | N150D | 5.09% | LHON, insulin resistance, AMD, NRTI-PN |
| MT-ND2 | G5460A | A331T | 5.93% | AD, Parkinson's disease |
| MT-ND2 | T5495C | *none* | 0.63% |  |
| MT-CO1 | C7028T | *none* | 77.72% |  |
| MT-ATP6 | G8697A | *none* | 4.90% | Thyroid tumor |
| MT-CO3 | G9477A | V91I | 4.35% | Thyroid tumor |
| MT-TR | T10463C | F20S | 4.95% | Endometrium tumor |
| MT-ND4 | A11251G | *none* | 9.73% |  |
| MT-ND4 | A11467G | *none* | 13.34% | Altered brain pH |
| MT-ND4 | G11719A | *none* | 74.34% |  |
| MT-ND4 | A11812G | *none* | 3.55% |  |
| MT-TL2 | A12308G | K15E | 13.27% | CPEO, stroke, cancers (CM breast, renal, prostate, lung, prostate, lung, prostate), altered brain pH, endometrium tissue |
| MT-ND5 | G12372A | *none* | 14.27% | Altered brain pH, prostate tumor |
| MT-ND5 | G12771A | *none* | 0.97% |  |
| MT-ND5 | G13368A | *none* | 5.20% |  |
| MT-ND5 | T13617C | *none* | 4.28% |  |
| MT-ND6 | A14233G | *none* | 3.89% |  |
| MT-CYB | C14766T | T7I | 73.82% |  |
| MT-CYB | A14793G | H16R | 2.42% |  |
| MT-CYB | G14905A | *none* | 5.42% |  |
| MT-CYB | A15218G | T158A | 2.10% |  |
| MT-CYB | A15326G | T194A | 98.72% |  |
| MT-CYB | C15452A | L236I | 9.72% |  |
| MT-CYB | A15607G | *none* | 5.37% | Breast tumor |
| MT-TT | A15924G | T13A | 3.74% | Lethal infantile mitochondrial myopathy |
| MT-TT | G15928A | G14E | 5.12% | Multiple Sclerosis, idiopathic repeat miscarriage, and AD protection |
| Control region | T16126C | Non-coding | 11.68% | Glioblastoma |
| Control region | C16184T | Non-coding | 0.65% |  |
| Control region | C16222T | Non-coding | 0.79% |  |
| Control region | T16231C | Non-coding | 1.00% |  |
| Control region | A16235G | Non-coding | 0.59% |  |
| Control region | C16256T | Non-coding | 3.68% |  |
| Control region | C16270T | Non-coding | 5.29% |  |
| Control region | C16291T | Non-coding | 2.79% |  |
| Control region | C16294T | Non-coding | 9.01% |  |
| Control region | T16304C | Non-coding | 6.61% | Esophageal, breast and prostate tumors |
| Control region | A16399G | Non-coding | 2.65% |  |
| Control region | T16519C | Non-coding | 61.92% | Glioblastoma, gastric & lung & ovarian & prostate tumor |

Abbreviations used are: Mt, mitochondrial; GB, gene bank; freq, frequency; PEO, progressive external ophthalmoplegia; MNGIE, mitochondrial neurogastrointestinal encephalopathy disease; LHON, Leber’s hereditary optic neuropathy; AMD, age-related macular degeneration; GDM, gestational diabetes mellitus; LVNC, left ventricular noncompaction; NRTI-PN, nuclease reverse transcriptase inhibitor associated peripheral neuropathy; CPEO, chronic progressive external ophthalmoplegia; CM, cardiomyopathy; HCM, hypertrophic cardiomyopathy; DMDF, diabetes mellitus+deafness; AD, Alzheimer’s disease

*The gene bank (GB) frequency data is derived from 26850 GeneBank sequences with size greater than 14kbp (www.mitomap.org)
