# Supplementary material for: Detection of Ultra-Rare Mitochondrial Mutations in Breast Stem Cells by Duplex Sequencing
Source: PLoS One. 2015 Aug 25;10(8):e0136216. doi: 10.1371/journal.pone.0136216 (PMC4549069; doi:10.1371/journal.pone.0136216)
Supplement: S3 Table — (DOCX) [file pone.0136216.s011.docx]

**S3 Table.** New variants within the common and unique mutations identified between non-stem and stem cells.

New rare variants

| Mt gene | DNA variant | Amino acid change |
| --- | --- | --- |
| A. Common variants in all sets of non-stem cells from three women | | |
| **RNR1** | **C1518T** | **H291Y** |
| **TN** | **A5705C** | **T17P** |
| ND4L | C10547G | *None* |
| B. Common variants in all sets of stem cells from three women | | |
| **RNR1** | **G1476A** | **V277M** |
| ATP6 | A8577C | *None* |
| D. Variants found only in all sets of non-stem, but not in stem cells | | |
| ND4L | C10547G | *None* |
|  |  |  |

New low-heteroplasmic variants

| Mt gene | DNA variant | Amino acid change |
| --- | --- | --- |
| A. Common variants in all sets of non-stem cells from three women | | |
| **ND1** | **A3511C** | **T69P** |
| B. Common variants in all sets of stem cells from three women | | |
| **ND1** | **A3447C** | **Q47H** |
| **ATP8** | **A8512C** | **K49N** |
