# Supplementary material for: Detection of Ultra-Rare Mitochondrial Mutations in Breast Stem Cells by Duplex Sequencing
Source: PLoS One. 2015 Aug 25;10(8):e0136216. doi: 10.1371/journal.pone.0136216 (PMC4549069; doi:10.1371/journal.pone.0136216)
Supplement: S4 Table — (DOCX) [file pone.0136216.s012.docx]

|  | Whole mtDNA | Within mtDNA coding regions | | | | | | | | Among mutations occurring in mtDNA coding regions | | |
| --- | --- | --- | --- | --- | --- | --- | --- | --- | --- | --- | --- | --- |
| Sample ID | Total sequenced DCS Nts | No. of Syn. | No. of **Misse** | | No. of **Truncat** | | | No. of **Nonsyn** | | % Syn | | **% Nonsyn** |
| **HME 11** |  |  |  |  | | |  | | |  |  | |
| Non-stem | 19389999 | 61 | 175 | 14 | | | 189 | | | 24.4 | 75.6 | |
| Stem | 19262440 | 57 | 143 | 5 | | | 148 | | | 27.8 | 72.2 | |
| **HME 30** |  |  |  |  | | |  | | |  |  | |
| Non-stem | 14625056 | 45 | 127 | 7 | | | 134 | | | 25.1 | 74.9 | |
| Stem | 15316422 | 41 | 95 | 17 | | | 112 | | | 26.8 | 73.2 | |
| **HME 31** |  |  |  |  | | |  | | |  |  | |
| Non-stem | 37240333 | 88 | 264 | 15 | | | 279 | | | 24 | 76 | |
| Stem | 40754403 | 76 | 271 | 23 | | | 294 | | | 20.5 | 79.5 | |
| **Pooled** |  |  |  |  | | |  | | | 24.5 ± 0.3 | 75.5 ± 0.3 | |
| Non-stem |  |  |  |  |  |  |  |  |  |  |  |  |
| Stem |  |  |  | | |  | | |  | 25.0 ± 2.3 | 75.0 ± 2.3 | |

**S4 Table.** Distribution of nonsynonymous mutations of non-homoplasmic variants in the whole mtDNA and within mtDNA coding regions.

Abbreviations used are: Nts, nucleotides; DCS, duplex consensus sequences; mt, mitochondria; mut, mutation; Nonsyn, nonsynonymous mutation; Misse, missense mutation; Trucat, truncating mutation; Syn, synonymous mutation. The numbers of nonsynonymous mutations are sums of missense and truncating (nonsense) mutations. Pooled data from three women (ID# 11, 30, and 31) in percentages are mean ± S.E.M. (n=3).
