# Supplementary material for: Detection of Ultra-Rare Mitochondrial Mutations in Breast Stem Cells by Duplex Sequencing
Source: PLoS One. 2015 Aug 25;10(8):e0136216. doi: 10.1371/journal.pone.0136216 (PMC4549069; doi:10.1371/journal.pone.0136216)
Supplement: S5 Table — (DOCX) [file pone.0136216.s013.docx]

**S5 Table.** The number of non-synonymous mutations of non-homoplasmic variants in protein coding genes.

|  |  | HME 11 | | | | HME 30 | | | | HME 31 | | | | Pooled (HME 11, 30, and 31) | | | |
| --- | --- | --- | --- | --- | --- | --- | --- | --- | --- | --- | --- | --- | --- | --- | --- | --- | --- |
|  |  | Non-stem | | **Stem** | | Non-stem | | **Stem** | | Non-stem | | **Stem** | | Non-stem | | **Stem** | |
|  | DNA bases | Truncat | Nonsyn | **Truncat** | **Nonsyn** | Truncat | Nonsyn | **Truncat** | **Nonsyn** | Truncat | Nonsyn | **Truncat** | **Nonsyn** | Sum of Truncat | Sum of Nonsyn | **Sum of Truncat** | **Sum of Nonsyn** |
| ND5 | 1812 | 0 | 20 | **0** | **19** | 2 | 24 | **2** | **13** | 0 | 40 | **3** | **39** | 2 | 84 | **5** | **71** |
| CO1 | 1542 | 1 | 19 | **0** | **9** | 1 | 7 | **2** | **13** | 2 | 28 | **2** | **31** | 4 | 54 | **4** | **53** |
| ND4 | 1378 | 3 | 23 | **0** | **12** | 1 | 10 | **0** | **3** | 2 | 26 | **2** | **31** | 6 | 59 | **2** | **46** |
| CYB | 1141 | 2 | 13 | **0** | **7** | 1 | 11 | **1** | **11** | 0 | 22 | **2** | **30** | 3 | 46 | **3** | **48** |
| ND2 | 1042 | 0 | 6 | **2** | **8** | 1 | 8 | **0** | **6** | 1 | 16 | **2** | **13** | 2 | 30 | **4** | **27** |
| ND1 | 956 | 0 | 11 | **0** | **11** | 0 | 13 | **0** | **7** | 1 | 17 | **0** | **17** | 1 | 41 | **0** | **35** |
| CO3 | 784 | 0 | 7 | **0** | **12** | 0 | 4 | **2** | **7** | 0 | 11 | **2** | **17** | 0 | 22 | **4** | **36** |
| CO2 | 684 | 0 | 7 | **0** | **6** | 0 | 7 | **2** | **7** | 1 | 16 | **0** | **9** | 1 | 30 | **2** | **22** |
| ATP6 | 681 | 0 | 12 | **0** | **5** | 0 | 4 | **0** | **3** | 0 | 12 | **0** | **7** | 0 | 28 | **0** | **15** |
| ND6 | 525 | 0 | 4 | **0** | **3** | 0 | 2 | **1** | **1** | 3 | 5 | **1** | **9** | 3 | 11 | **2** | **13** |
| ND3 | 346 | 0 | 7 | **0** | **5** | 0 | 8 | **2** | **4** | 1 | 11 | **0** | **6** | 1 | 26 | **2** | **15** |
| ND4L | 297 | 0 | 4 | **0** | **6** | 0 | 2 | **0** | **0** | 2 | 10 | **1** | **5** | 2 | 16 | **1** | **11** |
| ATP8 | 207 | 0 | 5 | **0** | **2** | 0 | 3 | **1** | **4** | 0 | 2 | **1** | **3** | 0 | 10 | **2** | **9** |
|  | Sum | 6 | 138 | **2** | **105** | 6 | 103 | **13** | **79** | 13 | 216 | **16** | **217** | 25 | 457 | **31** | **401** |
| Pearson's correlation | |  | ***0.862*** |  | ***0.811*** |  | ***0.757*** |  | ***0.760*** |  | ***0.943*** |  | ***0.950*** |  | ***0.932*** |  | ***0.954*** |

Abbreviations used are: Nts, nucleotides; DCS, duplex consensus sequences; mt, mitochondria; mut, mutation; Nonsyn, nonsynonymous mutation; Trucat, truncating mutation. The numbers of nonsynonymous mutations are sums of missense and truncating (nonsense) mutations.
